# A computer simulation analysis of the accuracy of partial genome sequencing and restriction fragment analysis in estimating genetic relationships: an application to papillomavirus DNA sequences

**DOI:** 10.1186/1471-2105-5-102

**Published:** 2004-07-27

**Authors:** Baozhen Qiao, Ronald M Weigel

**Affiliations:** 1 Division of Epidemiology and Preventive Medicine, Department of Veterinary Pathobiology, University of Illinois, Urbana, IL 61801 USA

## Abstract

**Background:**

Determination of genetic relatedness among microorganisms provides information necessary for making inferences regarding phylogeny. However, there is little information available on how well the genetic relationships inferred from different genotyping methods agree with true genetic relationships. In this report, two genotyping methods – restriction fragment analysis (RFA) and partial genome DNA sequencing – were each compared to complete DNA sequencing as the definitive standard for classification.

**Results:**

Using the Genbank database, 16 different types or subtypes of papillomavirus were selected as study samples, because numerous complete genome sequences were available. RFA was achieved by computer-simulated digestion. The genetic similarity of samples, based on RFA, was determined from the proportion of fragments that matched in size. DNA sequences of four specific genes (E1, E6, E7, and L1), representing partial genome sequencing, were also selected for comparison to complete genome sequencing. Laboratory error was not taken into account. Evaluation of the correlation between genetic similarity matrices (Mantel's r) and comparisons of the structure of the derived dendrograms (partition metric) indicated that partial genome sequencing (for single genes) had higher agreement with complete genome sequencing, achieving a maximum Mantel's r = 0.97 and a minimum partition metric = 10. RFA had lower agreement, with a maximum Mantel's r = 0.60 and a minimum partition metric = 18.

**Conclusions:**

This simulation indicated that for smaller genomes, such as papillomavirus, partial genome sequencing is superior to restriction fragment analysis in representing genetic relatedness among isolates. The generalizability of these results to larger genomes, as well as the impact of laboratory error, remains to be demonstrated.

## Background

Precise estimation of genetic relatedness between isolates of a microorganism is important for determination of phylogenetic relationships, which has important applications in studies of disease transmission [1,2]. The definitive standard for assessing genetic relatedness among organisms is the complete genome sequence of nucleotide bases [3]. However, nucleotide sequencing is expensive and time-consuming, thus, generally it is impractical for use in most investigations, particularly when a large number of samples is analyzed.

Currently, one genotyping technique used frequently as an alternative to complete genome sequencing is restriction fragment analysis (RFA), in which restriction endonuclease enzymes cleave the genome at specific sites, producing DNA fragments that are then separated by size using electrophoresis [4]. The percentage of fragments matching in size has been commonly used as an index to represent the genetic similarity between samples [5,6]. The accuracy of RFA in determining the true genetic relationships can be influenced by several factors, including the number of restriction enzymes used, the specific enzymes selected for DNA digestion, and laboratory conditions [7-9].

Another common alternative to complete genome sequencing is partial genome sequencing, i.e., the nucleotide sequencing of a particular gene or segment of the genome [8,10]. The gene or genome segment is often targeted by polymerase chain reaction (PCR). Selection of an appropriate gene or region for analysis is critical for accurately representing phylogenetic relationships [11,12].

In a comparison of RFA and partial genome sequencing with respect to their similarity in interpreting a disease outbreak caused by pseudorbabies virus in a swine producing region in Illinois, USA, both genotyping methods generated similar conclusions about patterns of spread of the virus [13]. However, the accuracy of each genotyping method in representing the complete genome was not evaluated.

Restriction fragment analysis detects genetic variation by surveying specific endonuclease restriction sites over the entire genome; in contrast, partial genome sequencing detects genetic variation by comparing nucleotide bases from a specific region of the genome. Each method detects a different dimension of genetic variation, and each can detect only a proportion of the genetic variation present in the entire genome. Therefore, it is important to determine which method, using partial information, provides a more accurate estimation of genetic relatedness.

The primary purpose of this study was to compare both restriction fragment analysis and partial genome sequencing to complete genome sequencing, with regard to their agreement in estimating genetic relationships and in reconstructing phylogenies under the ideal conditions of absence of laboratory error. Computer simulation of the genotyping analysis was conducted, using completely sequenced papillomavirus isolates obtained from Genbank.

## Results

**Figure 1:**
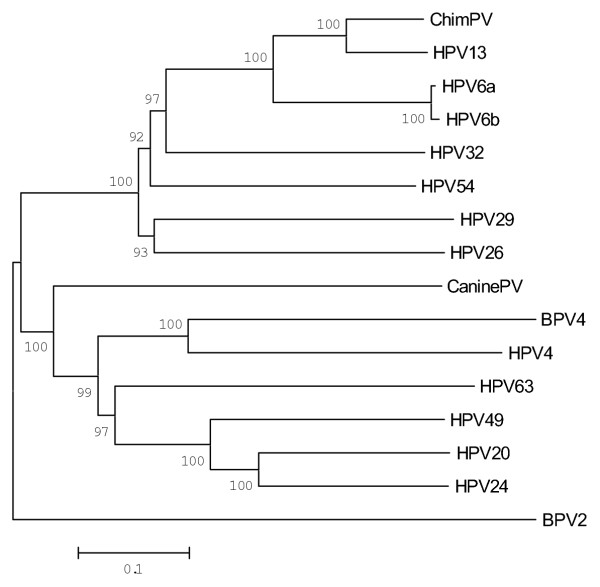
Tree of phylogenetic relationships among Papillomavirus samples, based on complete genome sequences. Classification achieved using the Neighbor-joining algorithm. The tree was rooted at the midpoint between the most disparate samples. Numbers on branches indicate bootstrap values.

**Figure 2:**
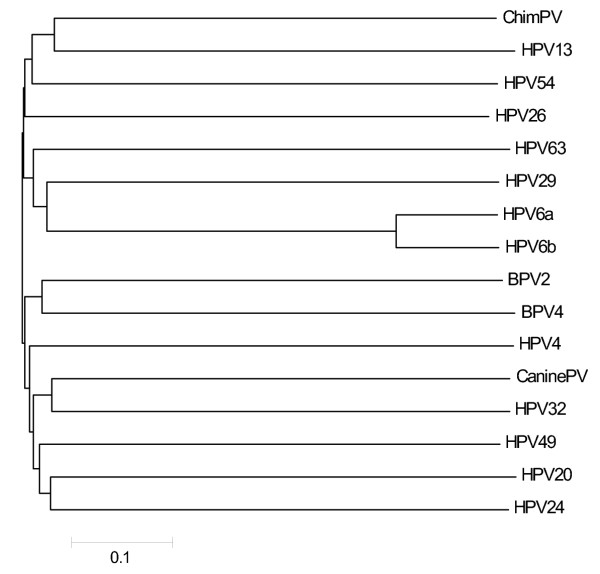
Tree of phylogenetic relationships among Papillomavirus samples, based on restriction fragment analysis with four restriction enzymes. Classification achieved using the Neighbor-joining method. The tree was rooted at the midpoint between the most disparate samples.

**Figure 3:**
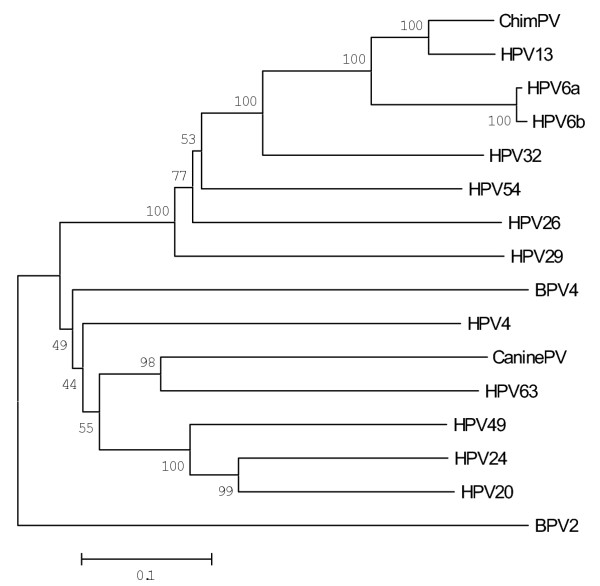
Tree of phylogenetic relationships among Papillomavirus samples, based on DNA sequencing of the E1 gene. Classification achieved using the Neighbor-joining method. The tree was rooted at the midpoint between the most disparate samples. Numbers on branches indicate bootstrap values.

Table 1 provides descriptive statistics on fragment size distributions for RFA (using the MaeI enzyme as an example) showing that a moderate number of fragments (mean > 20) were produced by simulated digestion. Fragment sizes were large (median ≈ 280 bps for example enzyme), with only 4 samples having one fragment each ≤ 20 bps. Table 2 shows that with an increase in the number of restriction enzymes, the correlation between the RFA and the complete genome sequencing genetic distance matrices increased slightly and the partition metric measuring dendrogram topological dissimilarity decreased slightly. The highest agreement with complete genome sequencing obtained for RFA was for a 4-enzyme combination, which achieved a maximum Mantel's r = 0.60 and minimum partition metric = 18. Table 3 shows that the similarity with complete genome sequencing in estimating genetic relatedness was much higher for partial genome sequencing, particularly for the E1 and L1 genes, which had the relatively longer sequences (averaging 24.2% and 19.6% of genome, respectively), although all genes selected had Mantel's r ≥ 0.88. The minimum value of the partition metric was 10, and the maximum value was 14, compared to a minimum of 18 for RFA. Phylogenetic trees are presented for complete genome sequencing (Fig. 1), RFA (the 4-enzyme condition with the highest agreement with complete genome sequencing) (Fig. 2), and sequencing of the E1 gene (the longest gene) (Fig. 3). Tree stability, as indicated by bootstrap values, was higher for complete genome sequencing (Fig. 1: all bootstrap values > 0.90) than for partial genome sequencing of the E1 gene (highest Mantel's r). However, the E1 gene tree structure for the most closely related samples was stable and nearly identical to complete genome sequencing. In contrast to the RFA example given (Fig. 2), which did not clearly differentiate the papillomavirus samples into subgroups, partial genome sequencing of the E1 gene identified 2 subgroups with the same composition (and BPV2 as an outlier) as did complete genome sequencing.

## Discussion

Sequencing entire genomes is impractical in most investigations of genetic relationships. The computer simulation conducted here determined that compared to restriction fragment analysis, partial genome sequencing had higher agreement with complete genome sequencing in estimating genetic relatedness and greater similarity in the topology of the dendrograms of phylogenetic relationships derived from these estimates. These results using papallomavirus sequences with a genome length averaging less than 8 kb, indicate that for microorganisms with small genomes, partial genome sequencing targeting genes comprising approximately 20–25% of the total genome length can provide a very good estimate of genetic relatedness. The topological structure of phylogenetic trees was also stable for partial genome sequencing, particularly for the most closely related samples. The degree to which these results generalize to larger genomes is unknown, in part because microorganisms with large genomes are rarely, if ever, sequenced in their entirety. There are also other considerations in selecting partial genome sequencing as a genotyping method, such as presence of the gene in all isolates, and sufficient variability to differentiate isolates [12]. In addition, whether genetic variation is random or due to natural selection needs to be taken into account [14], because in the latter case genetic dissimilarity may not reflect time since divergence, thus making it more difficult to infer evolutionary relationships, which are important for making inferences about pathogen transmission. These limitations should be considered as well for restriction fragment analysis.

One might expect that increasing genome size would diminish the advantage of partial genome sequencing compared to restriction fragment analysis. As total genome size increases, the number of restriction sites cut by restriction enzymes is expected to increase, providing more fragments and more genetic information for estimating genetic relatedness at no increased cost. This also needs to be taken into account in the selection of a genotyping method. However, it has been argued that if a gene is selectively neutral (i.e., variations are not subject to natural selection), it is only the length of the gene sequenced, not the ratio of sequenced gene length to genome size, that is important for determining the degree of divergence from a common ancestor [14]. To the extent that these conditions are satisfied, the results of this study indicate that specific gene sequencing is likely to provide a better estimate of genetic relationships than restriction fragment analysis of the complete genome under a wider variety of genome sizes.

The general conditions under which partial genome sequencing is more accurate than restriction fragment analysis in representing true genetic relatedness have not been addressed in the analysis conducted here. However, another study from our laboratory [15], using simulated genomes of various size with different nucleotide substitution rates, and varying degrees of genetic diversity among samples, found that only under conditions of both short partial genome sequence length and low rates of nucleotide substitution did RFA provide a more accurate topological reconstruction of phylogenetic relationships than did partial genome sequencing; the degree of genetic diversity among samples did not affect the advantage partial genome sequencing had in accurately depicting phylogenetic relationships. Thus, whether one is investigating the genetic relatedness among samples collected from a single disease outbreak or a diverse collection of samples from different times and geographic regions, under most conditions partial genome sequencing will represent genetic relationships more accurately than does RFA. Genotyping using partial genome sequencing and phylogenetic reconstruction (using the neighbor-joining algorithm) have become standard for several virus species, including not only papillomavirus [16,17], but also human immunodeficiency virus [18], classical swine fever virus [19], porcine reproductive and respiratory syndrome virus [20], and foot-and-mouth disease virus [21].

The simulated genotyping conducted here assumed no error of measurement. The sources of error in restriction fragment analysis are well known [22-24]. Fragments of similar size in the same lane of a gel may be indistinguishable, thus appearing to form one fragment. Fragments of small size may be undetectable. The relationship between migration distances and fragment size may be affected by variation in gel density both between and within gels. There are also differences in measurement error between laboratories [25,26]. These deficiencies are accounted for by use of marker DNA fragments of known nucleotide base pair length to assist in estimating cleaved DNA fragment sizes; however, acknowledgement of remaining error of measurement of the size of detectable fragments is inherent in the application of a tolerance range for considering fragments of similar but different sizes as a "match" [27]. Laboratory error is also inherent in partial genome sequencing [28]. With the commonly used polymerase chain reaction (PCR) methodology for detection and amplification of genes for sequencing, there can be error in primer development because primer sites may not be specific to the gene sequences or too specific to demarcate all occurrences of the gene. Heterogeneity of amplified DNA, due to replication error, recombination, low primer specificity, or impurity of the template can result in a failure to produce consistent sequencing results. In the comparison of the degree of similarity of DNA sequences between samples, alignment of sequences with unequal sequence lengths due to deletion or duplication, or the management of inverted sequences presents additional challenges for estimating genetic similarity and phylogenetic affinity [14]. The relative magnitude of sources of error in RFA versus partial genome sequencing is unknown and, thus, the conclusions presented here are those based upon the assumption of the absence or minimization of laboratory error.

In practical terms, laboratory error and cost need to be taken into account in the selection of a genotyping method. However, when the impact of these factors is minimized, the computer simulation analysis conducted here indicates that partial genome sequence becomes the preferred alternative for representing genetic relationships.

## Conclusions

For small genomes, partial genome sequencing of target genes comprising 20–25% of the total genome provides a more accurate estimate of genetic relatedness and more accurate representation of evolutionary and transmission histories than does restriction fragment analysis and thus is indicated to be the preferred genotyping method for phylogenetic reconstruction under these conditions. The degree to which these results are generalizable to larger genomes and conditions of laboratory error remains to be determined.

## Methods

### Sample DNA sequences

The source of information on nucleotide sequences was the Genbank database [29]. The organism selected for analysis was papallomavirus, for which a moderately large number of isolates with complete genome sequences was available. Human, bovine, canine, and chimpanzee papillomaviruses were considered. Among human papillomavirus (HPV) with complete genome sequencing available, 12 samples were selected at random: HPV 4, 6a, 6b, 20, 24, 49, 63, 13, 29, 32, 54, and 26. For bovine papillomavirus (BPV), complete genome sequences were available for BPV1, BPV2, and BPV4. Because the E1 gene of BPV1 (of interest for partial genome sequencing) could not be located, only BPV2 and BPV4 were chosen and included in the study. One type of canine oral papillomavirus (caninePV) and one type of common chimpanzee papillomavirus (chimPV) were available in the database, and these were chosen. Thus, a total of 16 types or subtypes of papillomaviruses that have been completely sequenced and stored in Genbank were used (Table 1).

The complete DNA sequences of the 16 papillomavirus samples were aligned using ClustalW software [30]. The genetic distances among these sequences were then calculated using the Kimura correction [31,32].

### Computer simulated restriction fragment analysis

#### Restriction endonuclease enzymes

Commonly used restriction endonuclease enzymes were selected [33], based on the following criteria: (1) Only enzymes with 4-base pair recognition sites were selected, in order to produce a sufficient number of fragments for analysis. (2) Among enzymes having the same recognition site, only one was selected. (3) For simplicity, enzymes with multiple recognition sites were excluded. Using these criteria, 15 restriction enzymes were included (AccII, AciI, AluI, BsuRI, CviRI, HapII, HhaI, MaeI, MaeI, MboI, MseI, NlaIII, RsaI, TaqI, TspEI).

#### Digestion

Simulated digestion of each papillomavirus DNA sample by each restriction enzyme was conducted using the DIGEST program [34]. The resulting restriction fragments for each sample were sorted by size (number of nucleotide base pairs).

#### Calculation of genetic distances

Based on the distribution of restriction fragment sizes, the genetic similarity between any two papillomavirus samples was calculated for each restriction enzyme using the Dice coefficient [5,6]: S_xy _= 2n_xy_/(n_x_+n_y_), where n_xy _is the number of fragments matching in size for samples x and y, and n_x _and n_y _are the number of fragments in samples x and y, respectively. Then, D_xy _= 1-S_xy _was calculated as a distance measure. Pairwise distances between samples were computed for each individual enzyme. Also, pairwise distances were obtained for up to 4 enzymes, by using for each condition (2, 3, and 4 enzymes) the fragment size distributions for 30 randomly selected combinations of enzymes, and calculating the composite distance [35].

### Partial genome sequence analysis

The E1, E6, E7, and L1 genes, which have been of interest in studies of papillomavirus, were used for estimating genetic relatedness. The ClustalW program [30] was used for sequence alignment, and the genetic distances (with the Kimura correction) were calculated for each gene.

### Agreement between genotyping methods

#### Correlation between distance matrices

The matrix of genetic distances based on complete DNA sequences was considered the definitive standard. The genetic distance matrices based on RFA and partial genome sequencing were compared to complete genome sequencing by calculating Mantel's coefficient of correlation between matrices (Mantel's r) [36].

#### Comparison of phylogenetic trees

The genetic distance matrices for RFA, partial genome sequencing, and complete genome sequencing were used to construct phylogenetic trees, using the Neighboring-joining algorithm [37], as implemented by MEGA software [38]. Trees were rooted at the midpoint between the most distantly related samples [39]. Bootstrap values indicating stability of tree topology were added to trees based on partial and complete genome sequencing [14]. The trees based on RFA and specific gene sequences were compared to the tree for complete genome sequencing, by using the COMPONENT software [40] to calculate the partition metric, which measures the difference in tree topology [41,42]. A lower value of partition metric indicates greater topological similarity.

## List of abbreviations

bps: base pairs

BPV: bovine papillomavirus

caninePV: canine papillomavirus

chimpPV: chimpanzee papillomavirus

HPV: human papillomavirus

kb: kilobase

Mantel's r: Mantel's coefficient of correlation between matrices

PCR: polymerase chain reaction

RFA: restriction fragment analysis

## Authors's contributions

BQ designed the investigation, collected the data, conducted the data analysis, and wrote the manuscript. RW identified the problem to be investigated, provided statistical guidance, assisted in interpretation of results, and edited the final drafts of the manuscript. Both authors read and approved the final manuscript.

## Figures and Tables

**Table 1:** Sequence lengths and fragment size distribution for papillomavirus samples obtained from Genbank

Type of Papillomavirus	Genbank Accession Number	Length of Complete Genome (bps)	Length of Genes (bps)				Fragment Size Distribution (digested by MaeI enzyme^1^)			
			
			E1 Gene	E6 Gene	E7 Gene	L1 Gene	Number of Fragments	Median Fragment Size (bps)	5% Percentile (bps)	95% Percentile (bps)
HPV4	X70827	7353	1800	422	303	1550	24	215	54	817
HPV6a	L41216	8010	1886	452	297	1502	18	249	43	1151
HPV6b	X00203	7902	1930	452	297	1502	17	207	47	1241
HPV20	U31778	7757	1818	497	309	1550	24	294	9	781
HPV24	U31782	7452	1824	422	291	1538	13	501	55	1354
HPV49	X74480	7560	1830	416	312	1529	18	232	24	1210
HPV63	X70828	7348	1857	425	267	1523	23	221	29	968
HPV13	X62843	7880	1941	452	306	1499	21	352	9	765
HPV29	U31784	7916	1983	446	273	1511	16	435	33	1003
HPV32	X74475	7961	1929	428	315	1511	21	276	36	881
HPV54	U37488	7759	1902	434	288	1493	28	158	20	815
HPV26	X74472	7855	1917	452	315	1511	20	341	47	877
BPV2	M20219	7937	1815	413	384	1493	27	204	17	809
BPV4	X05817	7265	1932	300	363	1562	19	323	31	750
CaninePV	D55633	8607	1794	434	294	1511	24	303	24	715
ChimPV	AF020905	7889	1947	458	300	1505	18	236	35	1504
**Mean**		**7778.1**	**1881.6**	**431.4**	**307.1**	**1518.1**	**20.7**	**284.1**	**32.2**	**977.5**

1: Data reported for MaeI as an example.

**Table 2:** Similarity of restriction fragment analysis to complete genome sequencing in estimating genetic relatedness between papillomavirus samples

Number of Enzymes	Mantel's r			Partition Metric		
	
	Mean	Standard Deviation	Maximum	Mean	Standard Deviation	Minimum
1	0.37	0.13	0.54	23.33	1.23	20
2	0.42	0.09	0.55	22.60	1.19	20
3	0.46	0.08	0.58	22.07	1.44	20
4	0.49	0.08	0.60	21.40	1.44	18

Mantel's r is the correlation between matrices of genetic similarity. The partition metric indicates topological similarity of dendrograms, with lower values indicating greater similarity.

**Table 3:** Similarity of partial genome sequencing to complete genome sequencing in estimating genetic relatedness between papillomavirus samples

Gene	Mantel's Correlation Coefficient	Partition Metric
E1	0.97	12
E6	0.92	10
E7	0.88	14
L1	0.96	12

Mantel's r is the correlation between matrices of genetic similarity. The partition metric indicates topological similarity of dendrograms, with lower values indicating greater similarity.
